# Framework for the evaluation of military health systems

**DOI:** 10.1136/bmjmilitary-2020-001699

**Published:** 2021-02-22

**Authors:** Ryan Leone, J Whitaker, Z Homan, L Bandekow, M Bricknell

**Affiliations:** 1 Conflict and Health Research Group, King's College London—Strand Campus, London, UK; 2 Department of Vascular Surgery, Royal Free Hospital NHS Trust, London, UK; 3 War Studies, King's College London—Strand Campus, London, UK; 4 Worldwide Military-Medicine Almanac, Bonn, Germany

**Keywords:** organisation of health services, International health services, public health, medical education & training

## Abstract

The organisation of a military health system (MHS) differs from the civilian system due to the role of the armed forces, the unique nature of the supported population and their occupational health requirements. A previously published review of the Military Medical Corps Worldwide Almanac demonstrated the value of a standardised framework for evaluation and comparison of MHSs. This paper proposes such a framework which highlights the unique features of MHSs not covered by health services research of national health systems. These include: national context and summary; organisational structure; firm base facilities, healthcare beneficiaries and medical research; operational capabilities, overseas deployments, collaborations and alliances; personnel including recruitment, training and education; and history and culture. This common framework can help facilitate international collaboration between military medical services including capability development, training exercises and mutual support during military operations. It can also inform national contributions to future editions of the Almanac.

## Introduction

**Figure 1:**
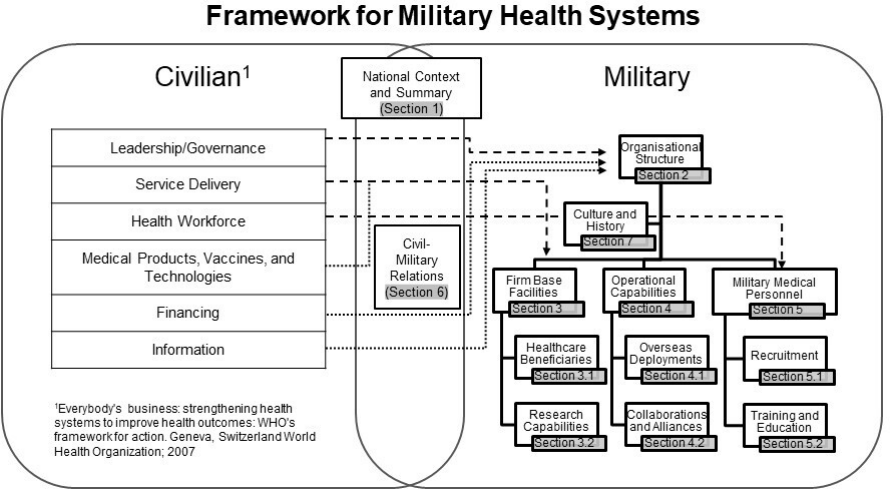
Framework for military health systems.

Two previous papers in this journal have highlighted the importance of understanding the relationships between the various providers of healthcare within a country, including the security sector and the armed forces, in order to inform Defence Health Engagement activities.^1 2^ Military medical personnel need to understand how their health system interfaces with their country’s wider health system and, if deployed overseas, the relationship between civil and military systems in the host nation. This requires a framework for the comparison of health systems, several of which already exist for the civilian context. International organisations like the World Health Organisation (WHO),^3^ Organisation for Economic Co-operation and Development (OECD)^4^ and non-profits like the Commonwealth Fund^5^ provide health profiles of different nations in order to inform policies for health systems reform. However, these comparisons do not cover military health systems (MHSs). Military healthcare providers may be a significant supplier of government-funded healthcare and a substantial component of national and international responses to global health crisis, including the ongoing fight against SARS-CoV-2.^6–8^ We recently published an analysis of the primary online summary of MHSs,^9^ the Military Medical Corps Worldwide Almanac (Almanac).^10^ We identified common features of MHSs and also highlighted the wide variation in breadth and depth of data in the Almanac on MHSs between countries. This paper proposes a framework for evaluating MHSs to improve mutual understanding for domestic and international collaboration between civilian and military healthcare organisations. It is summarised in Figure 1 and is matched to the WHO ‘6 Building Blocks of a Health System’. It has the following sections: national context and summary; organisational structure; firm base facilities, military-specific beneficiaries and medical research; operational capabilities, overseas deployments, collaborations and alliances; personnel including recruitment, and training and education; and history and culture. A blank framework is provided as an online supplemental file to this paper, including data tables to support each section.

10.1136/bmjmilitary-2020-001699.supp1 Supplementary data

### Section 1: national context and summary for military health systems

This section provides an overview to the unique characteristics that apply to the MHS that supports a country’s armed forces. The size and likely missions for the MHS will be dependent on the international and domestic security threats that determine the role of a nation’s armed forces. This sets the requirement for healthcare for military personnel outside of a nation’s borders in overseas garrisons and during military operations.^11^ This crucial role requires the rapid deployment of human and material resources, and demands that key elements of the MHS can be released from responsibilities for domestic healthcare provision. Beyond deployments, a MHS is primarily an occupational health service, maximising the medical fitness of service-members through health protection, prevention and treatment, and recovery for sick and injured service-members. This is similar to a civilian health system and this role will be determined by national public health services and social security arrangements. Depending on national circumstances, the MHS may also have discrete research, training, personnel management, healthcare information, logistics and pharmaceutical services. The MHS will normally compete for resources from the defence budget rather than the health or social security budget. Therefore, its value and cost are measured against its outputs for defence rather than health.

### Section 2: organisational relationships

**Figure 2:**
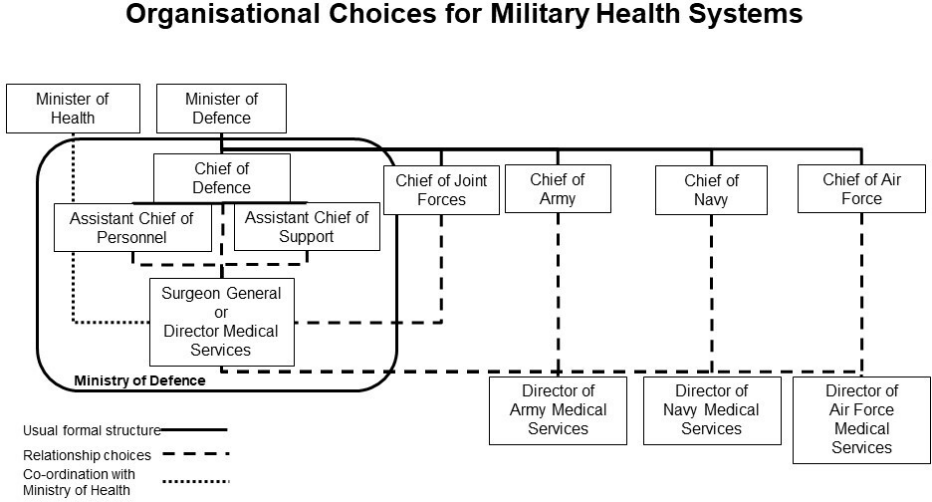
Organisational choices for military health systems.

This section covers leadership and governance of the MHS. It should include a structural diagram and a narrative to explain organisational relationships. The civilian system is ultimately under the jurisdiction of the Ministry of Health (MoH). However, MHSs are normally accountable to the Ministry of Defence (MoD). The relationship between the Chief of Defence and the head of the MHS is subject to variation between countries based on size and circumstances. Figure 2 shows the possible organisational choices for military health services, using filled lines to suggest the military structure, dashed lines to indicate choices about relationships and a dotted line to indicate the external connection from the military to the MoH. The diagram assigns the title of Surgeon General (SG) or Director Medical Services (DirMS) to the senior appointment in the MHS. This reflects the dual role of ‘senior health adviser to Defence’ as a ‘staff function’ (providing health intelligence, planning and logistical or operational health insights) to the Chief of Defence, and the ‘senior leader of the medical services’ as a ‘chief executive’ function (controlling operational medical units, garrison healthcare, training centres and research facilities). The choices indicated in the diagram reflect the range different governance models for a MHS. For example, the SG or DirMS may be directly subordinate to the MoD as a medical chief, as it is done in Germany, or subordinate to a joint chief as it is done in the UK.^9^ The SG or DirMS may also directly command the all the medical services that support that Army, Navy and Air Force (eg, Ireland and Israel), or coordinate between leaders of healthcare for each service (eg, Nigeria).

### Section 3: the firm base health system

This section covers community-based services within military garrisons (primary medical care, dental care, physical rehabilitation and mental health) and hospital services.^12^ This is often the largest component of the MHS and a vital part of the benefits of military service. The beneficiaries may dictate the breadth of clinical services provided (obstetrics to geriatrics) and the overall cost of the MHS.

#### Section 3.1: healthcare beneficiaries

This subsection provides a matrix to record the beneficiaries of the MHS. In addition to armed forces personnel, there may also be an obligation to provide general medical services to beneficiaries, such as family members, retirees, veterans and designated civilians.^12^ It may also care for law enforcement personnel or VIP patients, such as royalty and elected officials.^2^

#### Section 3.2: medical research and innovation

Military research has facilitated significant advances in healthcare from preventative medicine and vaccines, to acute trauma care and specialist rehabilitation.^13–15^ A nations’ military medical research capabilities and focus may be a significant additional component of the Firm Base health system. This subsection lists possible categories of research in the MHS, including whether there are physical military facilities for conducting this research, funding for relevant civilian sector research and military-specific medical journals.

### Section 4: operational capabilities

This section covers the role of a MHS to treat and evacuate casualties from military operations from point of injury through different levels of care back to the home nation. Medical units may be codified according to the capability descriptions in NATO Allied Joint Medical Support Doctrine^16^ and used to inform national and international military capability planning such as the NATO Defence Planning Process and the EU Capability Development Priorities.^17 18^ A description might be supported by a graphic that illustrates the national casualty evacuation concept (eg, Taiwan and Thailand).^9^ It is recognised that a full description of the number and size of military medical units might be classified.

#### Section 4.1: overseas and operational Deployments

This subsection covers the participation of the MHS in overseas combat missions, training exercises, disaster relief operations and UN peacekeeping operations. To understand the scale of such commitments, it is suggested that the annual number of each of these three deployment types is categorised by the number of missions and the level of resources committed each year. Additionally, the section could include a map to illustrate the breadth of their commitments (eg, Jordan and Israel).^9^

#### Section 4.2: collaborations and alliances

Military medicine is often a significant topic for international collaboration such as coordination of joint exercises in combat or disaster scenarios, presentation of research and production of common clinical standards. This subsection also covers the participation of the MHS in multinational medical collaborations such as the International Committee on Military Medicine and regional congresses,^19^ the NATO Centre for Military Medicine^20^ and the Balkan Military Medical Committee.^21^

### Section 5: military medical personnel

This section covers the healthcare workforce which is a key building block of the MHS and is the principal cost. The WHO’s mapping of healthcare occupations, based on the 2008 International Standard Classification of Occupations, combines armed forces providers under ‘armed forces occupations’.^22^ This obscures the detail necessary to understand a MHS’s capability. There are three unique personnel aspects of MHSs. The first is that the ‘combat medic’ (service-members trained to deliver preventative, primary and combat casualty care) is often a unique military job. Though similar to paramedics or clinical officers, their training and employment may not align with civilian categories of healthcare providers.^23^ The second is the employment status of the personnel. Military systems may include active-duty service-members, reservists or civilian personnel, with implications for their availability to work on overseas operations or domestically. The third is that serving military providers will have a military rank as either officer or enlisted. This may have implications for healthcare team structures and dynamics. A suggested list of professional categories is included in the online supplemental file.

#### Section 5.1: military medical recruitment

Armed forces compete in the national market for health professionals and many nations have difficulty recruiting to their full requirement. This subsection lists a range of recruitment ‘incentives’ from direct conscription, financial inducements, sponsored education, MOD-delivered professional education and access to non-military income that might be used to improve recruiting and retention in the MHS.

#### Section 5.2: military medical training and education

This subsection covers the arrangements for the two requirements for career development of military health personnel: military training and healthcare professional education. This might be delivered through separate institutions: military training centres providing generic skills to the standards of the armed forces; military medical training centres that provide the unique medical skills needed for the military environment;^24^ and professional educational pathways aligned to civilian competencies and qualifications. There may also be a specific training centre for military medical units.

### Section 6: civil–military relations

This section covers formal relationships between the civilian and MHSs. The COVID-19 crisis has highlighted the contribution of military medical services to national crisis response. Military healthcare efforts may intersect with civilian efforts to improve trauma care, coordinate disaster response^25^ and facilitate the provision of subspecialty care that is beyond the capability of military facilities.

### Section 7: history and culture

The final section covers the cultural and historical features of a MHS that create a sense of unity, identity and loyalty.^26^ The shared symbols of military culture, like badges, mottos and associations, may influence the perspectives of military healthcare providers. Additionally, the history of a nation’s MHS may be a source of pride and a foundation for traditions, ceremonies, and rituals. This might be recorded in books or displayed within a military medical museum.

### Limitations

There is potential for deeper international understanding of MHSs if this framework is used to record key features and data of a MHS. However, some nations may limit the description of their military medical operations and capabilities due to security concerns. Additionally, it is important to recognise that this framework concentrates on military-specific capabilities and that it does not capture health outcomes and other measures of health system responsiveness. This framework has been developed from ‘best practice’ information provided by nations for the Almanac. It may require further review if the Almanac receives more complete country profiles.

## Conclusion

This paper proposes a framework to capture the unique features of MHSs. This framework can support comparative analysis between MHSs and facilitate cooperation and interoperability within national health systems. It might also provide a structure for national entries within the Almanac. The section headings and key information is shown in Table 1. A fuller description of each section and a blank framework is available as an online supplemental file to this paper.

**Table 1:** Summary of the framework for military health systems

Section	Title	Summary
1	National context and summary for military health systems	Brief description of the country, its military system and its military health system.
2	Organisational relationships	Leadership and governance of the MHS.
3	The firm base health system	Community-based services within military garrisons (primary medical care, dental care, physical rehabilitation and mental health) and hospital services.
3.1	Healthcare beneficiaries	List of all beneficiaries of the MHS for example, armed forces personnel, families, retirees, VIPs and so on.
3.2	Medical research and innovation	Organisations and relationships for research in military healthcare and armed forces personnel.
4	Operational capabilities	Capabilities of the MHS to treat and transfer casualties from military operations from point of injury through different levels of care back to the home nation.
4.1	Overseas and operational deployments	Breadth and scale of overseas/operational commitments.
4.2	Collaborations and alliances	Participation of the MHS in international healthcare collaborations and alliances.
5	Military medical personnel	Professional categories and numbers of personnel in the MHS (including civilians).
5.1	Military medical recruitment	Method of recruiting personnel for the MHS, including scholarships and other incentives.
5.2	Military medical training and education	Arrangements for career development of military health personnel: military training and healthcare professional education.
6	Civil–military relations	Arrangements for collaboration between the civilian and military health systems, including in crisis.
7	History and culture	Cultural and historical features of a MHS that create a sense of unity, identity and loyalty.

MHS, military health system.
