# Supplementary material for: Framework for the evaluation of military health systems
Source: BMJ Mil Health. 2021 Feb 22;169(3):280–4. doi: 10.1136/bmjmilitary-2020-001699 (PMC10313955; doi:10.1136/bmjmilitary-2020-001699)
Supplement: Supplementary data [file bmjmilitary-2020-001699supp001.pdf]

The Military Health Services of [insert country]

Section 1: National Context and Summary

This section provides an overview to the unique characteristics that apply to healthcare for a country's armed forces. Insert a brief description of the country, its military system and its military health system (MHS). This should include any unique characteristics of the wider national health system that impact on the military medical system. **Please include answers to the following questions.**

- How are the armed forces organised?
- Where are the main organisations located?
- Does the military and its medical services operate as Joint or Single Services?
- How are health and social care financed? Does the military replicate this for armed forces personnel, families and retirees?
- How is healthcare information managed across the civilian and military health system?

Section 2: Organisational Structure

This section covers leadership and governance of the MHS. Insert an organisational diagram plus a supporting narrative to illustrate the relationship between the Surgeon General function (technical health advice to the Ministry of Defence, and health policy direction to the army, navy and air force) and the Director General function (chief executive role over components of the military health system). **Please include answers to the following questions.**

- What is command and organisation structure of the MHS?
- Who does the head or heads of the military health system report to?
- What are the main medical formations and units at the strategic, operational and tactical levels and where are they located?
- Does the military use contractors to provide support in the firm base and on operations?
- How are financial resources allocated?

Section 3: Firm Base Health System

Leone R, *et al. BMJ Mil Health* 2023; 169:280–284. doi: 10.1136/bmjmilitary-2020-001699

This section covers the Firm Base health system (see: Bricknell M, Cain P. 'Understanding the Whole of Military Health Systems - The Defence Healthcare Cycle,' *The RUSI Journal*. 2020. https://www.tandfonline.com/doi/full/10.1080/03071847.2020.1784039). This comprises community health services (family practice, occupational health, dental care, mental health, sports medicine/rehabilitation, community hospitals) and hospital services (either military hospitals or facilitated access to public/private hospitals). **Please include answers to the following questions.**

- What is the size of Firm Base health system?
- Is there a map showing the distribution of medical facilities?
- Are there any special military medical treatment capabilities? E.g. rehabilitation centre, mental health centre, cancer centre etc
- What is the relationship between military Firm Base healthcare and the civilian public health system?
- How is military medical/clinical information exchanged and managed in the Firm Base?

Section 3.1 – Healthcare Beneficiaries

This sub-section provides a matrix to record the beneficiaries of the MHS. In addition to armed forces personnel, there may also be an obligation to provide general medical services to beneficiaries, such as family members, retirees, veterans and designated civilians. Table 1 provides a suggested categorisation.

Table 3.1 - Beneficiaries of the Firm Base Military Health System.

**Beneficiary**	**Definition**	**Number**	**Remarks**
Active Duty	Full-time members of the Army, Navy, Air Force		
Other Security Forces	Personnel who serve in counter-terrorism teams, the gendarmerie, or other law enforcement representatives.		
Reserve	Non-full-time members of the Army, Navy, Air Force		Might be different categories of reserves
Family Members	Relatives of Active Duty members of the Armed Forces		Might include spouses, children, parents or other relatives

Leone R, *et al. BMJ Mil Health* 2023; 169:280–284. doi: 10.1136/bmjmilitary-2020-001699

Beneficiary	Definition	Number	Remarks
Retirees	Personnel who have left Active Duty employment		
Veterans	Personnel who have left Active Duty employment with a medical condition attributable to military service		
Government Civilians	Personnel who work for the military and/or government as civilians.		
VIP Populations	Personnel who have privileged royal, elected, or otherwise important status who receive care from the military without qualifying due to other factors.		
Public Civilians	Members of the general public not fitting any of the above categories		This might be provided free or require co-payment

Section 3.2: Military Medical Research and Innovation

Medical research for the military environment is an important component of a military health system and is often linked to military medical education institutions. Table 2 lists categories of potential military medical research. This sub-section should include a description and link to any national academic journals in military medicine. **Please include answers to the following questions.**

- Does the military have physical infrastructure in place to research each topic?
- Are there civilian institutions and researchers who are funded by the military to conduct this research?
- Are there joint research projects conducted across the civilian and military spheres?
- Does the nation publish any academic journals in military medicine?

Table 3.2: Categories of Research Conducted by a Military Health System

Research Category	Description of Research Capability
Aviation and Aerospace	

**Research Category**	**Description of Research Capability**
Diving, Underwater and Naval	
Tropical and Infectious Disease	
Public Health	
Chemical, Biological, Radioactive, and Nuclear	
Mental Health	
Technology and Telemedicine	
Rehabilitation and Prosthetics	
Combat Casualty Care	
Human Performance in Austere and Extreme Environments (heat, cold)	

Section 4: Operational Capabilities

This section covers the role of a MHS to treat and transfer casualties from military operations from point of injury through different levels of care back to the home nation. The narrative might be supported by a graphic that illustrates the national military casualty evacuation system. The NATO capability definitions (Table 3) may be a helpful framework, though nations may combine more than one role into a single medical unit (regiment or battalion). There may be security constraints that prevent recording the precise numbers and capacity of medical units. **Please include answers to the following questions.**

- Does the military have any standing domestic and international military tasks?
- What is the operational capability and capacity?
- Are there capabilities for damage control surgery and in-theater surgery?
- What are the types of forward, tactical and strategic MEDEVAC capabilities and their capacities?
- How are new medical equipment or capabilities procured?

Leone R, *et al. BMJ Mil Health* 2023; 169:280–284. doi: 10.1136/bmjmilitary-2020-001699

- How is medical logistics organised and managed during operations?

Table 4: Medical Operational Capabilities (see: Allied Joint Medical Support Doctrine AJP-4.10. North Atlantic Treaty Organization Standardization Office. 2019. At: https://coemed.org/files/stanags/01_AJP/AJP-4.10_EDC_V1_E_2228.pdf)

**Capability**	**Definition**	**Numbers**	**Comment or Description**
Role 1	Triage, pre-hospital emergency care, essential diagnostics, and limited holding capabilities.		
Role 2F	Mobile and deployable structures that may perform damage control resuscitation and damage control surgery in far-forward or unsecured environments.		
Role 2B	Mobile and deployable structures that may perform damage control resuscitation and damage control surgery.		
Role 2E	Mobile and deployable structures that may perform damage control resuscitation and damage control surgery, along with expanded capabilities that may include x-ray equipment, blood banks, pharmaceutical supplies,, and sterilization equipment.		
Role 3	Deployable hospital and specialist care that incorporates CT technology and oxygen production.		
Role 4	Full-spectrum capabilities outside of the deployed environment that include reconstructive surgery, rehabilitation, and other specialized techniques.		
Casualty Staging Units	Patient holding centers with nursing care that may hold and stabilize patients before transport between levels of care.		
Medical Emergency Response Team	Pre-hospital care teams that can provide care in non-combat operational environments.		
Forward Evacuation	Transportation from the point of injury to an initial medical treatment facility.		

Leone R, *et al. BMJ Mil Health* 2023; 169:280–284. doi: 10.1136/bmjmilitary-2020-001699

Capability	Definition	Numbers	Comment or Description
Tactical Evacuation	Transportation from one medical treatment facility to another within the area of operations.		
Strategic Evacuation	Transportation from medical facilities within the area of operations to medical facilities outside of the area of operations.		
Maritime Evacuation Assets	Sea-based vehicles that may evacuate individuals from maritime or amphibious operations.		
Land Evacuation Assets	Ambulances that can transport casualties over difficult terrain.		
Air Evacuation Assets	Tilt rotor, rotary, or fixed wing assets such as helicopters and planes that may transport patients through the air.		

Section 4.1: Overseas or Operational Deployments

This section should indicate the breadth and scale of overseas/operational commitments by the military medical services. This might be illustrated on a map and supported by data entered into Table 4. **Please include answers to the following questions.**

- How many combat operations, disaster relief missions, and UN Peacekeeping Operations has the nation completed this year?
- What current medical operations are being conducted by the nation's military abroad?

Table 4.1: Operational Deployments

Resource Commitment	Combat Operations	Disaster Relief	UN Peacekeeping Operations
Individual Teams			
Role 1 Involvement			

Role 2 Involvement			
Role 3 Involvement			

Section 4.2: Collaborations and Alliances

This section summarises the participation of the MHS in international healthcare collaborations and alliances. Please list the alliances that the nation takes part in and fill out the table below. **Please include answers to the following questions.**

- Is the nation a member of any multi-national military medical organisations?
- Who are the nation's closest collaborators during operational and training missions?

Section 5: Military Medical Personnel

This section covers key aspects of military medical personnel management, training and recruitment. It should provide numbers of military personnel with a narrative that highlights key aspects such as: military medical technicians, extended training for military roles (e.g. nurse anaesthetists), and the balance between the active duty, reserve, and civilian workforce. **Please include answers to the following questions.**

- What is the total number of personnel in each the medical services of the Army, Navy, Air Force, civilian?
- What is breakdown of professions/specialities?
- What is the role of reservists and civilians in the military medical system?

Table 5: Categories of Healthcare Personnel in a Military Medical System

Leone R, *et al. BMJ Mil Health* 2023; 169:280–284. doi: 10.1136/bmjmilitary-2020-001699

**Personnel Type**	**Definition**[1]	**Active Duty #**		**Reservist #**		**Civilian #**	**Total #**
**Rank**		**Officer**	**Enlisted**	**Officer**	**Enlisted**	**N/A**	
Physicians	Clinicians who have obtained medical degrees with the proper licensing and training to practice in general medicine or in specialized disciplines.[15]						
Veterinarians	Certified clinicians who diagnose, treat, and prevent diseases in animals.[15]						
Dentists	Clinicians who are licensed to treat diseases of the mouth, teeth, jaws, and related areas.[15]						
Pharmacists	Professionals who are licensed to store and distribute medications.[15]						
Nurses	Clinicians with nursing degrees and credentials to provide care to patients.[15]						
Combat Medics	Clinical care providers who are trained by the military, but are not necessarily provided with the certification levels and education required for physician and non-physician clinician roles.[13]						
Healthcare Administrator	Any non-clinical personnel, such as NCO practice managers and medical support officers, who help with the management of domestic and international healthcare facilities.[28]						
Allied Health Professional	Personnel who support physicians and non-physician clinicians, including dental hygienists, physical therapists, medical technicians, radiographers, speech pathologists, respiratory therapists, among others.[29]						
Non-Physician Clinicians	Licensed clinical practitioners who are not certified physicians, including nurse practitioners, mid-wives, physician assistants, chiropractors, naturopaths, acupuncturists, optometrists, and podiatrists, among others.[30]						

[1] See: World Health Organization. Classifying health workers: Mapping occupations to the international standard classification. *World Health Organization,* 2010. At; https://www.who.int/hrh/statistics/Health_workers_classification.pdf?ua=1

Section 5.1: Military Medical Recruitment

Leone R, *et al. BMJ Mil Health* 2023; 169:280–284. doi: 10.1136/bmjmilitary-2020-001699

This sub-section should provide information about the recruitment pathways for military medical providers. **Please include answers to the following questions.**

- How are physician and non-physician providers recruited to the military?
- Which groups receive their primary professional training in the military? (doctors, nurses, AHPs, non-physician clinicians)

Table 5.1: Recruiting Mechanisms for Military Health Professionals

Recruiting Mechanism	Definition	Presence
Monetary	Physicians are offered monetary bonuses or higher compensation than civilian alternatives would provide.	
Scholarship or Repayment	Physicians are offered scholarships for schooling while in training or repayment programs after their training is completed.	
Mandatory Service	Physicians in the country are required to serve in the military.	

Section 5.2: Military Medical Training and Education

Many nations have institutions for the training and education of military healthcare personnel including field training centres, nurse and AHP training centres, military medical schools and collaborations for post-graduate training of healthcare professionals. These should be recorded in this section, including civil-military collaboration. **Please include answers to the following questions.**

- How are basic soldiering skills and medical specialist training delivered?
- Is there mandatory individual training in military skills such as weapon handling, CBRN protection and first aid?
- Are there specific military medical training institutions? E.g. field training centre, military medical school, military nursing school etc?
- How is the military collectively trained and validated to meet readiness?

Table 5.2: Education and Training Systems for Military Health Professionals

Training Mechanism	Definition	Presence
Civilian Education	Students pursue their education at a civilian university with no military component.	
Military Education	Students pursue their professional qualifications at a military university/training centre.	

Leone R, *et al. BMJ Mil Health* 2023; 169:280–284. doi: 10.1136/bmjmilitary-2020-001699

Military Track Education	Students pursue their medical degree at a civilian university that possesses a military faculty and pathway.	
Foreign Military Education	Students pursue their education at a military medical school in a foreign, but allied, country.	

Section 6: Civil-military relations

This section covers formal arrangements for civil-military collaboration in health services and research. **Please include answers to the following questions.**

- Are there formal arrangements for co-ordination/collaboration between civilian and military health systems?
- Are the military health services included within national disaster risk reduction and resilience planning?
- Are there examples of military medical services being used as part of national crisis response?

Table 6: Examples of categories of Civil-Military Relationships

**Activity**	**No**	**Yes - Briefly describe**
Civil-military cooperation in a domestic trauma system		
Military providers working in a civilian context when not deployed		
Joint civil-military disaster operations domestically		
Joint civil-military disaster operations abroad		
Integration of military and civilian garrison hospitals		

Section 7: History and Culture

This section should summarize the historical and cultural of the military health system, tracing from its early stages to the current status of the military health system (see: Bricknell M. The Cultural Challenge of Leading in Military Medicine. *Military-medicine.com.* 2020. Retrieved from https://military-medicine.com/article/3839-the-cultural-challenge-of-leading-in-military-medicine.html). **Please include answers to the following questions.**

- When did the nation's military health system begin?
- What changes has the military health system undergone over time?

Leone R, *et al. BMJ Mil Health* 2023; 169:280–284. doi: 10.1136/bmjmilitary-2020-001699

- What symbols and cultural structures have become shared across all military providers?

Table 7: Examples of cultural artefacts/symbols/features of a MHS

Category	Definition	Description
History	A narrative description of the initial founding and subsequent development of the military health system.	
Military badges or emblems	A badge/emblem that denotes an association with the MHS.	
Mission Statement or Motto	A statement of purpose or short phrase that defines the function of the military health system.	
Military Medical Associations	Organizations that facilitate community and socialization between military providers either during or after their service.	
Museums	Facilities that describe the history of a nation's military healthcare and store relics of the system's past.	
Historical Books	Literary descriptions of the historical development of a nation's military healthcare system.	
Websites	Links to any websites that provide information about a nation's past or present military health system.	

Leone R, *et al. BMJ Mil Health* 2023; 169:280–284. doi: 10.1136/bmjmilitary-2020-001699
